# Neural basis of implicit cognitive reappraisal in panic disorder: an event-related fMRI study

**DOI:** 10.1186/s12967-021-02968-2

**Published:** 2021-07-13

**Authors:** Hai-Yang Wang, Guo-Qing Xu, Ming-Fei Ni, Cui-Hong Zhang, Xue-Lin Li, Yi Chang, Xiao-Pei Sun, Bing-Wei Zhang

**Affiliations:** 1 Department of Neurology, Jining No. 1 People’s Hospital, Jining, 272000 China; 2 Department of Neurology and Psychiatry, The First Affiliated Hospital of Dalian Medical University, No.222, Zhongshan Road, Dalian, 116011 Liaoning Province China; 3 Department of Psychology, Dalian Medical University, Dalian, 116044 China; 4 Department of Radiology, The First Affiliated Hospital of Dalian Medical University, Dalian, 116011 China; 5 Department of Geriatric Medicine, Huizhou Third People’s Hospital, Guangzhou Medical University, Huizhou, 516000 China; 6 Department of Intensive Care Unit, Jining No. 1 People’s Hospital, Jining, 272000 China

**Keywords:** Panic disorder, Emotion regulation, Implicit cognitive reappraisal, fMRI, Prefrontal cortex

## Abstract

**Background:**

Panic disorder (PD) is thought to be related with deficits in emotion regulation, especially in cognitive reappraisal. According to the cognitive model, PD patients’ intrinsic and unconscious misappraisal strategies are the cause of panic attacks. However, no studies have yet been performed to explore the underlying neuromechanism of cognitive reappraisal that occur on an unconscious level in PD patients.

**Methods:**

Twenty-six patients with PD and 25 healthy controls (HC) performed a fully-verified event-block design emotional regulation task aimed at investigating responses of implicit cognitive reappraisal during an fMRI scan. Participants passively viewed negatively valanced pictures that were beforehand neutrally, positively, or adversely portrayed in the task.

**Results:**

Whole-brain analysis of fMRI data showed that PD patients exhibited less activation in the right dorsolateral prefrontal cortex (dlPFC) and right dorsomedial prefrontal cortex (dmPFC) compared to HC, but presented greater activation in parietal cortex when negative pictures were preceded by positive/neutral vs negative descriptions. Simultaneously, interactive effects of Group × Condition were observed in the right amygdala across both groups. Furthermore, activation in dlPFC and dmPFC was is negatively correlated to severity of anxiety and panic in PD when negative images were preceded by non-negative vs negative descriptions.

**Conclusions:**

Emotional dysregulation in PD is likely the result of deficient activation in dlPFC and dmPFC during implicit cognitive reappraisal, in line with impaired automatic top-down regulation. Correlations between severity of anxiety and panic attack and activation of right dlPFC and dmPFC suggest that the failure to engage prefrontal region during implicit cognitive reappraisal might be associated wtih the severity of anxiety and panic; such functional patterns might be the target of possible treatments.

## Background

Emotional regulation is defined as an attempt to influence the emotions of oneself or others [1]. Negative emotions caused by adverse events need to be regulated to avoid interfering with ongoing activities and long-term goals [2]. Successful control of unpleasant emotion is critical to an individual’s well-being, health, and psychological and social functioning [3]. Emotion dysregulation (difficulties in effectively managing one’s emotions) is closely associated with the onset, maintenance, and therapy of various types of anxiety disorders [4].

Panic disorder (PD) is an anxiety disorder characterized by the recurrence of spontaneous panic attacks with psychological, physical, and functional conditions [5]. Clinically, emotion dysregulation is considered to be a pivotal aspect in the pathophysiological mechanism of anxiety and mood disorders and is a hallmark of PD [6, 7]. Indeed, cognitive emotion dysregulation as an important mechanism in PD is increasingly receiving attention [8–10].

Based on the emotion regulation model proposed by Gross [11], cognitive reappraisal, the most extensively explored skill is one type of antecedent-focused emotion regulation strategy that alters the trajectory of emotional responses by changing the meaning of the situation [12]. Reinterpretation and distancing are two cognitive reappraisal tactics [13]. Reinterpretation is described as altering one's interpretation of the stimulus or situation that provokes the emotion, whereas distancing is described as altering one’s personal or psychological distance from the stimulus or situation that elicits emotion. Cognitive reappraisal is remarkably effective in emotion regulation strategies [11] and results in enduring effects [13]. Decreased use of cognitive reappraisal strategies explains some catastrophic interpretations of anxiety-inducing conditions (for example, “catastrophic thinking”), which serves a critical role in the theory of cognition in PD [14, 15]. Based on PD’s cognitive model, panic is attributed to catastrophic misappraisal of bodily sensations [16], referring to patients’ tendencies to catastrophically misunderstanding psychosomatic responses that are associated with reduced use of cognitive reappraisal, which can act as a conditioning stimulus to trigger and sustain panic [17]. Emotion regulation strategies have been measured using emotion regulation questionnaires; for instance, our previous research showed that PD patients may involve emotion dysregulation associated with cognitive reappraisal [18]. We found that PD patients use fewer positive reappraisal but more catastrophic strategies compared to healthy controls [18]. Therefore, it has crucial theoretical and practical significance to explore the neural mechanisms of cognitive emotion dysregulation in patient with PD.

Besides cognitive reappraisal, another emotion regulation strategy involving Gross’s emotion regulation model is particularly relevant to PD is expressive suppression, a less well studied. Expressive suppression is a response-focused maladaptive strategy that involves consciously inhibiting behavioral responses to emotions [3]. Patients with PD commonly attempt to hide their anxiety symptoms by using maladaptive expressive suppression strategy to control their psychosomatic responses, fearing others may notice them, which is unsuccessful and frequently contributes to additional deterioration of symptoms [19]. While some evidence suggests that patients with PD use more expressive suppression strategy compared to the healthy controls [9, 20], one study failed to detect this difference [21]. Thus, there is no consistent conclusion about the differences in the use of expressive suppression between PD and healthy control compared to cognitive reappraisal.

The process of emotional regulation can be either explicit (conscious) or implicit (unconscious) [22, 23]. In clinical practice, panic attacks are suddenly and sometimes unexpectedly paroxysmal bursts of severe anxiety [24]. Catastrophic misappraisal can be subconsciously manipulated, such as during panic attacks while sleeping or when particular catastrophic notions are unrecalled [24], and there is usually no conscious cognitive reappraisal process before or during panic attacks. Studies that have examined the mechanisms of anxiety psychopathology show that the emotional regulation difficulties in anxiety are essentially due to deficient engagement of implicit emotion regulation strategies [25]. The catastrophic cognition hypothesis states that PD patients unconsciously evaluate internal and external stimuli that are inadequate to trigger a violent response as threat signals, consequently triggering panic attacks [16]. Recent studies have found that PD patients demonstrated an anomalous mismatch negativity of acoustic and visual (emotional and non-emotional) stimuli, implicating anomalous implicit information processing in patients with PD [26, 27]. Therefore, we speculate that it is likely that PD patients’ intrinsic and implicit cognitive reappraisal strategies are responsible for triggering panic attacks and that PD-related abnormal emotional regulation is not only manifested in the realm of consciousness but likely more in implicit reappraisal mechanisms.

Over the last decade, studies investigating the neural mechanism that contribute to emotion dysregulation in PD have shown consistent hypo-activation in prefrontal regions involving the dlPFC, dmPFC, and ventrolateral prefrontal cortex during cognitive reappraisal [8, 9]. Patients in these researches were asked to interpret the aversive stimuli as less negative deliberately and consciously through reinterpretation to reduce their unpleasant emotional experiences. Thus, the extra effort and cognitive control might indicate the possibility that participants had to deliberately produce their substitute explanations for the aversive pictures. However, these studies do not effectively reveal the neural basis of cognitive reappraisal that occurs out of consciousness in PD.

Zhang et al. adopted an implicit reappraisal paradigm to explore the neural basis and time processes in unconscious reappraisal in PD with event-related potentials (ERPs) [10, 28]. In Zhang’s study, PD patients and healthy controls received a brief neutral or negative description before viewing the negative pictures. During passive viewing of adverse pictures, cognitive emotion regulation processes were effortless, uninstructed, and unconscious [23]. The results showed that the negative pictures pre-described with neutral descriptions exhibited decreased late positive potential (LPP) amplitudes than the negative pictures pre-described with a negative description in healthy subjects. In contrast, no reliable effect of description condition was found for LPP in the PD patients. These results demonstrate an impairment in the regulatory process of cognitive reappraisal in PD [10]. Moreover, the task used is a reactive reappraisal paradigm that avoids the effects of increased task effort or additional underlying emotional processes, providing further proof that reduced LPP modification supports aberrant implicit cognitive reappraisal in PD patients [28]. ERP can effectively reflect the time course of brain electrical activity, but the low spatial resolution means that it can be difficult to pinpoint the precise localization of functional neural correlates. In contrast, fMRI has high spatial resolution and is the most effective method to study neural functioning. However, to our knowledge, there have been no fMRI studies regarding the nerve mechanism of implicit cognitive reappraisal in PD.

Therefore, the current study was designed to explore the neural basis of implicit cognitive reappraisal in PD by using neuropsychological assessments, self-report measures of emotional experience, and fMRI. The reappraisal tactics for emotion regulation mainly include reinterpretation and distancing [29]. Reinterpretation was used as a cognitive reappraisal strategy in this research based on previous studies [9, 23]. Behavioral data and fMRI data were recorded from PD and HC using the implicit cognitive reappraisal paradigm developed by Foti and Hajcak [30]. This task involves two conditions: (a) negative images were pre-described with non-negative (neutral or positive) descriptions (implicit reappraisal condition) and (b) negative images were pre-described with negative descriptions. Studies in implicit emotion regulation of healthy populations have shown that implicit emotion regulation can effectively decrease negative valence ratings of subjects, and increase activity in prefrontal regions such as dlPFC and dmPFC, the parietal lobe, and other brain regions, accompanied by decreased activity in limbic systems [31, 32]. In earlier work, we investigated the neural bases of implicit emotion regulation in healthy subjects by using the implicit cognitive reappraisal paradigm and demonstrated that implicit reappraisal processes could recruit prefrontal areas involving the dlPFC, dmPFC, and parietal cortex to modulate emotional responding [23]. On the basis of these results, we hypothesized that PD patients would not be able to automatically adjust their emotional state through implicit cognitive reappraisal strategy when receiving neutral/positive descriptions before the negative affective pictures. Moreover, we expected that the PD group would report negative feelings and correspondingly show decreased activity in prefrontal regions involving dlPFC and dmPFC compared to healthy controls. This would reflect the greater affective responsiveness to adverse stimuli and the lack of automatic inhibition of emotional responses to adverse stimuli in PD. Given that the biological model points to inadequate cognitive control as a factor in generating panic [23, 33], we further hypothesized that emotion disorder in PD might be the result of inadequate top-down control during implicit reappraisal.

## Materials and method

### Participants

Participants were between the ages of 18 and 65, right-handed, were Chinese Asians (Han nationality, in China only), without current and past major medical or neurological conditions, and certified by two board-certified physicians. Since this is the first investigation to explore implicit cognitive reappraisal in PD, it is unable to determine the required sample size based on a priori power analysis. Therefore, we set the desired sample size to 26 in the PD group with reference to two recent fMRI studies in PD that explore deliberate cognitive reappraisal strategy [8, 9]. Twenty-six untreated patients met criteria for clinically predominant PD based on Diagnostic and Statistical Manual of Mental Disorders, 5th Edition (DSM-5) [5] and 25 HC without DSM-5 axis-I history were recruited from the emergency and outpatient departments of the First Affiliated Hospital of Dalian Medical University and the surrounding communities. After providing written informed consent, participants completed the Hamilton Anxiety Rating Scale (HAM-A) [34], Hamilton Depression Rating Scale (HAM-D) [35], the Panic-Associated Symptom Scale (PASS) [36], Panic Disorder Severity Scale (PDSS) [37], and Cognitive Emotion Regulation Questionnaire (CERQ) [38]. Individuals were informed of the safety and eligibility criteria for fMRI scan: no cognitive impairment (dementia, traumatic brain damage, mental deficiency, and instrumental brain syndrome), no other neurological impairment, and no contraindications to fMRI (e.g., implanted ferrous metal, pregnancy, claustrophobia). Considering the variability in the treatment process, all subjects were free of any antipsychotic drugs for at least two weeks (benzodiazepine intake were medication-free 48 h) before the scan. Owing to the high co-morbidity rate in PD and affective or other anxiety disorders [39], co-morbid mood and other anxiety disorders were permitted if the PD diagnosis was primary. In the PD group, two patients were combined with generalized anxiety disorder, four patients with social anxiety disorder, and three patients with depressive disorder. Table 1 presents the demographic characteristics of the 51 participants. Informed consent was obtained from all participants prior to participation according to the Declaration of Helsinki principles. This study was approved by the ethics committee of the First Hospital of Dalian Medical University.

**Table 1:** Demographics and clinical features of participants

	PD	HC	*F*_1,49_/χ2	*P* score
Gender (male/female)	13/13	12/13	χ2 = 0.20	0.89
Age (years)	35.6 (8.0)	35.2 (6.7)	0.033	0.86
Education in years	13.0 (3.1)	14.4 (2.0)	3.4	0.072
HAM-A	15.3 (6.3)	2.3 (2.1)	94.4	< 0.001***
HAM-D	10.8 (5.2)	3.5 (1.8)	4.29	< 0.001***
PDSS	10.3 (5.0)	–	–	–
PASS	8.5 (4.7)	–	–	–
CERQ scales				
Self-blame	6.2 (2.3)	5.3 (1.7)	2.25	0.140
Acceptance	7.3 (1.9)	6.48 (1.7)	2.50	0.120
Rumination	7.7 (1.6)	6.4 (1.8)	6.92	0.011*
Positive refocusing	4.8 (1.2)	5.3 (1.6)	3.87	0.055
Refocus on planning	7.1 (2.1)	7.5 (1.7)	2.95	0.092
Positive reappraisal	6.2 (1.6)	7.5 (1.4)	9.84	0.003**
Putting into perspective	5.6 (2.0)	6.9 (1.4)	9.40	0.004**
Catastrophizing	5.7 (2.0)	3.4 (1.3)	25.49	< 0.001***
Other-blame	5.0 (1.7)	4.6 (1.4)	0.57	0.453

*PD* panic disorder, *HC* healthy controls, *HAMA* Hamilton Anxiety Rating Scale, *HAMD* Hamilton Depression Rating Scale, *PDSS* Panic Disorder Severity Scale, *PASS* Panic-Associated Symptom Scale, *CERQ* Cognitive Emotion Regulation Questionnaire

^*^P < 0.05, **P < 0.01, ***P < 0.001

### Measures

The PASS is a nine-item scale used for evaluating the severity of a wide range of symptoms of PD [36]. The symptoms range from the frequency and intensity of situational panic attacks, the frequency and intensity of spontaneous panic attacks, the amount and intensity of anticipatory anxiety, to phobia-induced distress. The total scores range from 0 to 28. The Chinese version of the PASS scale was used, which has high internal consistency (Cronbach’s α = 0.72) and reliability (test-retest intraclass correlation coefficient = 0.64–0.77) [40].

The Panic Disorder Severity Scale (PDSS) is a seven-item scale to evaluate the overall severity of panic disorder [37]. The scale evaluates the frequency of panic attacks, distress during attacks, anticipatory anxiety, fearful avoidance, feelings of fearful avoidance, the impairment in functional ability at work, and the impairment in social functioning. It is a four-point Likert scale format with a total score between 0 and 28. The Chinese version of the PDSS scale has high internal consistency (Cronbach’s α = 0.74) and reliability (test-retest intraclass correlation coefficient = 0.70–0.89) [40].

The HAM-A is a 14-item questionnaire administered by clinicians to measure the severity of anxiety symptoms [34].Severity of depressive symptoms was measured using the validated 24-item Hamilton Depression Rating Scale (HAM-D) [35].

The CERQ was used to assess cognitive emotion regulation strategies after exposure to unpleasant life incidents [38]. The CERQ is composed of 9 distinct scales, all consisting of two items, including maladaptive strategies (i.e., rumination, self-blame, catastrophizing, and other-blame) and adaptive startegies (i.e., acceptance, positive refocusing, refocus on planning, positive reappraisal, and putting in perspective). Each item was scored on a 5-point Likert scale ranging from 1 (almost never) to 5 (almost always). The Chinese version of the CERQ has favorable reliability and validity with Cronbach’s α of 0.81 for the full scale, inter-item correlation coefficients of 0.1 for the full scale, and mean inter-item correlation coefficients ranging from 0.19 to 0.71 for each subscale [41].

### Stimuli and task

**Fig. 1:**
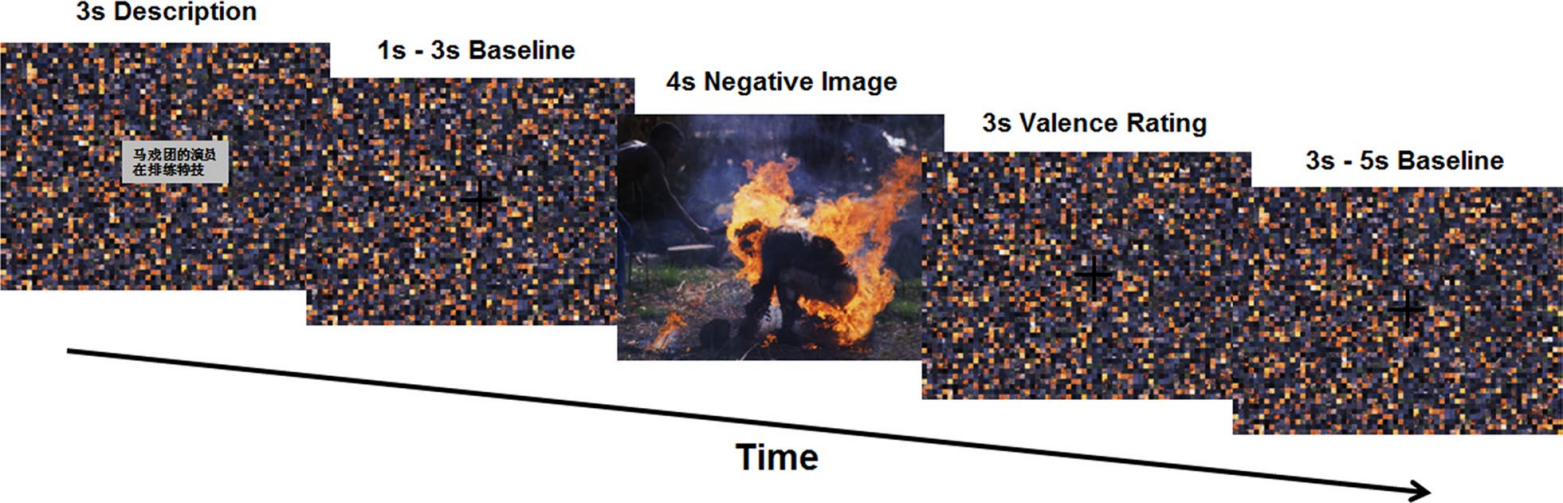
Experimental task design. The paradigm involves an antecedent-focused and implicit cognitive reappraisal manipulation. Each participant was presented with a neutral/positive or negative Chinese description of the upcoming picture that stayed on the screen for 3 s, accompanied with a scrambled picture and a fixed cross in black (1–3 s). The negative picture was then shown for 4 s and the participant looked at the picture passively (the period of interest). Following this, participants were again shown the scrambled picture and fixed crosse for 3 s and were asked to use the keyboard to assess their emotional valence from 1 (none too negative) to 4 (extremely negative). Scrambled picture with a fixed black cross continue to be presented subsequently (3–5 s). As a sample in this tril, subjects view the following neutral/positive words: *The actor from circus is rehearsing stunt*. Each trial was 16 s long, and 50 trials (25 neutral/positive and 25 negative descriptions) were randomly shown in two runs, and all images were displayed a single time

In the present study, reinterpretation rather than psychological distancing was used as the reappraisal tactic. Participants completed the implicit cognitive reappraisal paradigm developed by Wang et al. [23] during an MRI scan. The paradigm comprised two runs. Each run paradigm comprised 25 trials. The negative pictures were shown for 4 s in each 16-s trial, preceded and followed by the corresponding dislocated pictures (and fixation crosses in black) for a 12-s total (Fig. 1). In each run, a 3-s scrambled picture first appears on the screen, in which appears either a neutral/positive or negative depiction of the upcoming picture that is still present on the monitor. After 1–3 s (jittered) of presentation of the same scrambled picture with a fixed cross as a baseline, the negative picture was shown for 4 s and the participant looked at the picture passively. Following this, participants were again shown the dislocated pictures and fixed crosses for 3 s and were asked to use the keyboard to assess their emotional valence from 1 (none too negative) to 4 (extremely negative). The same dislocated picture with a black fixed cross was then presented for 3–5 s (jittered) as a baseline. Of interest to our hypothesis was the 4 s, during which subjects passively viewed unpleasant pictures.

We chose 60 negative pictures from the International Affective Picture System [42] as stimuli, using a pixel-level dislocated version of each picture as a baseline. The preceding descriptions of 25 pictures highlighted the negative sides of the pictures (negative descriptions preceding negative pictures condition, NEG-DESC), while the other 25 descriptions depicted pictures with a neutral or positive form (non-negative descriptions preceding negative pictures condition, NNEG-DESC). As a sample, subjects view the following negative descriptive words: *This man was burned alive*. The same picture’s neutral or positive descriptive words were: *The actor from circus is rehearsing stunt*. The aim of this operation was to investigate the differences in brain activation to negative pictures between the NNEG-DESC condition and NEG-DESC condition. In both runs, the order of trials and the corresponding negative or non-negative descriptions before each negative picture is randomized. All subjects were required to finish 25 NNEG-DESC trials and 25 NEG-DESC trials. Prior to the fMRI scan, participants finished 10 practice trials in which no pictures were used in experimental trials to confirm understanding. Before the task started, subjects were informed to simply watch and do not think otherwise. After the task, participants all reported that they were not aware of the intention of the task was emotion regulation before it started and after it ended and carefully followed the task requirements without doing active thoughts during the task.

### Behavioral data analysis

The behavioral data (i.e., four-point rating of valence) were analyzed using a 2 × 2 mixed measures analysis of variance (ANOVA), including the between-subject factors of group (PD, HC) and within-subject factors of condition (NNEG-DESC, NEG-DESC). Post hoc paired-samples t-tests were performed to investigate the presence and direction of group differences in negative affect valence ratings in each condition.

### Image acquisition

Images were obtained using 3.0-Tesla MR scanner (SignaHDx, GE Healthcare, Chicago, IL). A volume head coil was used for radiofrequency reception and transmission. Two fMRI runs sensitive to blood oxygenation level-dependent (BOLD) were performed, lasting 6 min each. The scanning parameters were as follows: TR/TE = 2000/30 ms, slice thickness/gap = 2.6/1.4 mm, slice number = 36, field of view (FOV) = 220 × 220 mm^3^, matrix size (per slice) = 64 × 64, flip angle = 90°. High-resolution T1-weighted anatomical images were collected using BRAVO sequence TR/TE = 8/1 ms, FOV = 256 × 256 mm^2^, flip angle = 12°, slice thickness = 1 mm, number of slices = 184, no gap, during the same session for normalization, co-registration, and data visualization.

### fMRI data analysis

Functional MRI data were pre-processed and analyzed using the Analysis of Functional NeuroImages (AFNI) software package [43]. To allow for magnetization equilibrium before image acquisition, the first four volumes of the functional images were discarded. All functional images were slice time corrected with reference to the first acquired slice. Then, a six-parameter rigid-body spatial transformation was used to spatially correct the image for head movement. The high-resolution anatomical images were then acquired for co-registration with the functional scans. Spatial smoothing of the normalized functional images was performed with an 8-mm FWHM Gaussian kernel. Finally, linear and quadratic trends were modeled for each voxel's time course to control.

Individual-level whole-brain general linear model analysis was conducted on the preprocessed fMRI data. The regressors of interest were NNEG-DESC and NEG-DESC conditions (i.e., corresponding dislocated picture with the fixed cross). Other regressors of non-interest included two regressors of participants viewing picture descriptions and six regressors head movement. The predicted activation time course was convolved with each subject's estimated hemodynamic response function modeled as a gamma probability density function.

Contrast maps for each subject were then resampled with the functional data resolution, normalized to Talairach coordinates. The main aim of our study was to separate emotional responses from implicit cognitive reappraisal; thus, in the presence of negative pictures during NNEG-DESC and NEG-DESC, neural activity was modeled separately, with each item contrasted with the baseline. In particular, the comparison of interest was NNEG-DESC and NEG-DESC. To examine the effects of interest, we carried out three t-tests across participants and produced the following t-maps: the condition effects in HC group ((NNEG-DESC)–(NEG-DESC)), the condition effects in PD group ((NNEG-DESC)–(NEG-DESC)), and the interactions between condition and group (HC ((NNEG-DESC)–(NEG-DESC))–PD ((NNEG-DESC)–(NEG-DESC))). Correction for multiple comparisons of contrast maps was performed in the whole brain level with the updated AFNI function 3dClustSim [44], which resulted in a whole-brain corrected probability of *p* < 0.05. We set the threshold at 0.05 for corrected results and got the cluster size of 162 through 3dClustSim. Cross-subject alignment [45] was not used as the implicit cognitive reappraisal paradigm was verified [23, 30] and the current task had some limitations (e.g., number of trials).

Considering the abundant evidence for amygdala involvement in the encoding of emotional memories and in the perception and labeling of unpleasant stimuli [13], we defined two spherical regions-of-interest (ROI) (radius = 6 mm). Peak voxels of the bilateral amygdalae were selected according to previous studies [12, 44]. We converted these coordinates used for ROI selection, given in the literature in MNI space, to Talairach space by a nonlinear transform. These ROIs (right amygdala coordinates: x = 24, y = − 3, z = − 13; left amygdala: x = − 16, y = − 6, z = − 10) were obtained from previously published studies. After extracting mean beta values from these ROIs of the NEG-DESC and NNEG-DESC conditions from each participant, values were entered into ANOVA to explore main effects and interactions.

Using Pearson’s correlations, we conducted a post hoc analysis to investigate a possible relationship between self-reported valence ratings and standardized betas (extracted from significant clusters) in the condition of NNEG-DESC, NEG-DESC, and NNEG-DESC vs NEG-DESC within PD and HC groups. To explore the potential connections between important clusters of activation during cognitive reappraisal (NNEG-DESC versus NEG-DESC) and symptom severity obtained from questionnaires mentioned above, we also calculated Pearson correlation between mean values extracted from significant clusters and HAM-A, HAM-D, PASS, PDSS, CERQ (18 items) in the PD group. Relationships between the questionnaire measures and average brain activation were examined for the brain regions (dlPFC, dmPFC, and parietal cortex) that differed between PD and HC groups and were associated with explicit and implicit cognitive reappraisal [12, 23]. Considering the amygdala is the core neural substrate of emotion processing, involved in the automatic processing of unpleasant stimuli (especially the perception of anger and fear expressions) and the implicit cognitive reappraisal [23, 46], beta estimates were extracted from the amygdala to investigate the correlation with questionnaire measures.

## Results

### Demographics and clinical features

Table 1 summarizes the demographics and clinical features of PD and HC groups. No remarkable differences were found between the PD and HC groups in sex, age, and years of education. One-way ANOVA demonstrated a major effect that was significant of group on HAM-A and HAM-D (*p* < 0.001). In the CERQ scale, scores relating to catastrophizing and rumination were considerably greater in the PD group than in the HC group, and scores relating to positive reappraisal and putting into perspective were lower in the PD group than in the HC group.

### Behavioral task effects

**Fig. 2:**
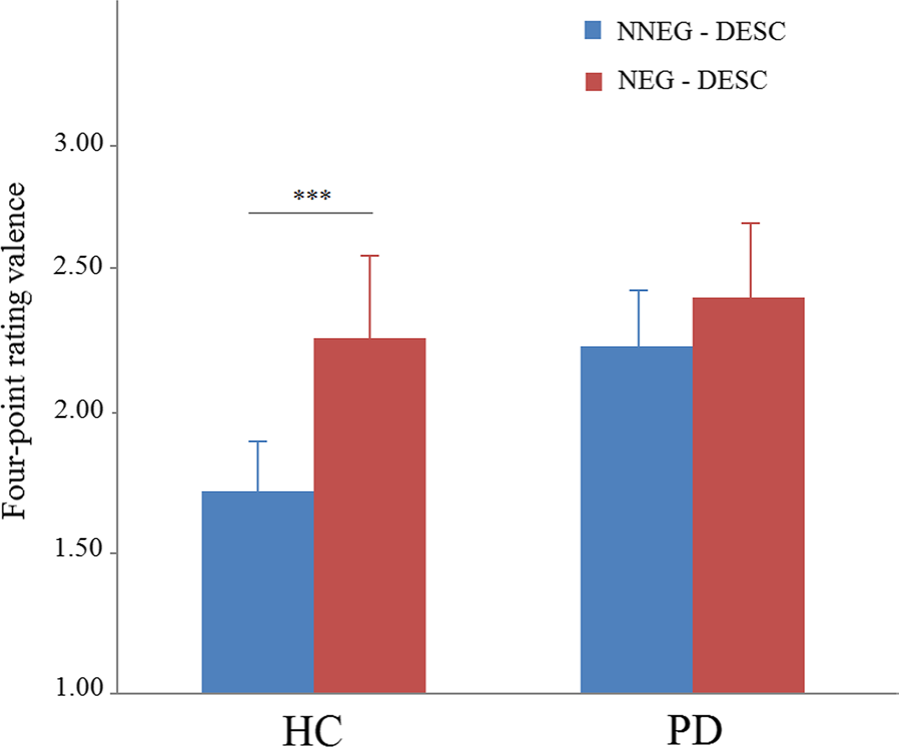
Negative emotion valence ratings during the task. Asterisks indicate HC group demonstrated significant difference in valence ratings between the NNEG-DESC and NEG-DESC conditions (***p < 0.001). In PD group, however, no differences in valence ratings between the NNEG-DESC and NEG-DESC conditions were found. HC, healthy control; PD, panic disorder

Results of the valence ratings are presented in Fig. 2. The ANOVA revealed a significant main effect condition (*F*_1,49_ = 38.75, *p* < 0.001, η^2^ = 0.39), group (*F*_1,49_ = 7.22, *p* = 0.010, η^2^ = 0.14), and a Condition × Group interaction (*F*_1,49_ = 22.19, *p* < 0.001, η^2^ = 0.24). Post hoc comparisons were performed using simple effect analysis showed significant differences in ratings of the NNEG-DESC and NEG-DESC conditions across the HC group (*F*_1,24_ = 28.53, *p* < 0.001), but there were no significant differences found in the PD group (*F*_1,25_ = 0.38, *p* = 0.544).

### fMRI task effects

**Fig. 3:**
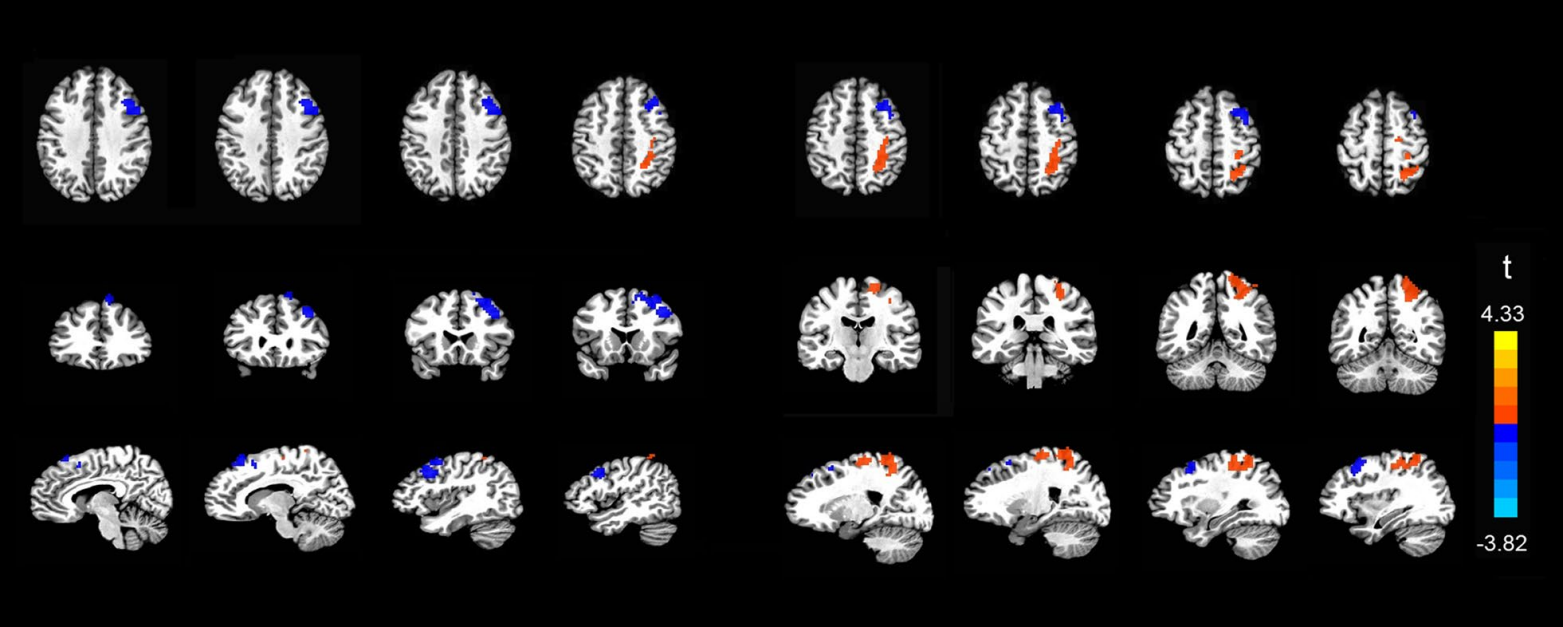
Whole-brain analysis. Group × task interaction shows that the PD group, compared to HC, exhibits less activation (blue color) in the right dlPFC and right dmPFC and more activation (orange color) in right parietal cortex during implicit cognitive reappraisal (NNEG-DESC *vs* NEG-DESC). Activations were thresholded at *p* < 0.05, corrected. PD, panic disorder; HC, healthy control

Results of the whole-brain voxel-wise analysis are shown in Table 2 and Fig. 3. We converted any coordinates in Talairach space reported to MNI space. In the HC group, effects of condition were observed over broad areas including bilateral frontal gyrus, bilateral temporal gyrus, and bilateral parietal gyrus. In contrast, the PD group showed these effects only observed over left frontal and parietal gyrus. Interaction effects were obtained over right parietal cortex (postcentral gyrus, precuneus, and superior parietal lobule, BA 7), and right dlPFC (BA 9) extending to dmPFC (BA 8). Two samples t-test analyses showed that the PD group exhibited less activation (blue color in Fig. 3) in the right dlPFC and right dmPFC in the NNEG-DESC vs NEG-DESC condition, compared to HC. The PD group also exhibited more activation (orange color in Fig. 3) in right parietal cortex during the NNEG-DESC condition compared to HC, but not in the NEG-DESC condition.

**Table 2:** Areas of significant decrease and increase in BOLD response in patients and controls during implicit cognitive reappraisal (NNEG-DESC *versus* NEG-DESC)

	Brain regions	BA	Side	Cluster size	MNI coordinates			T-value
				(voxels)	x	y	z	
PD group: NNEG-DESC > NEG-DESC	Precentral Gyrus/Postcentral Gyrus	4/6	L	1876	− 40	− 20	58	6.04
	Cerebellum	–	R	691	18	− 86	− 40	4.63
HC group: NNEG-DESC > NEG-DESC	Postcentral Gyrus/Precentral Gyrus/Inferior Parietal Loule	3/7/40	R	1371	47	− 29	58	− 5.92
	Dorsolateral prefrontal cortex/Dorsomedial prefrontal cortex	6/8/9	L	771	− 43	20	41	5.8
	Lateral Orbitofrontal Cortex	10/11	L	523	− 40	43	− 22	4.56
	Cerebellum	–	R	432	15	− 80	− 37	5.21
	Angular Gyrus/Precuneus/Inferior Parietal Lobule	19/39	R	409	40	− 74	48	4.90
	Middle Temporal Gyrus/Inferior Temporal Gyrus	20/21	R	399	62	− 44	− 13	4.34
	Postcentral Gyrus/Precuneus/Superior parietal lobule	7	R	366	37	55	− 23	4.81
	Angular Gyrus/Middle Temporal Gyrus/Precuneus	19/39	L	271	− 40	− 65	44	4.64
Interaction effects: HC(NNEG-DESC > NEG-DESC) > PD (NNEG-DESC > NEG-DESC)	Postcentral Gyrus/precuneus/Superior parietal lobule	7	R	295	20	− 51	66	4.33
	Dorsolateral prefrontal cortex ext	6/9	R	230	11	27	60	− 3.82
	Dorsomedial prefrontal cortex	8	R		9	19	51	− 3.22

*PD* panic disorder, *HC* healthy control, *ext* extending into, *BA* Brodmann area, *L* left, *R* right

x y z = MNI coordinates of the peak active voxel

**Fig. 4:**
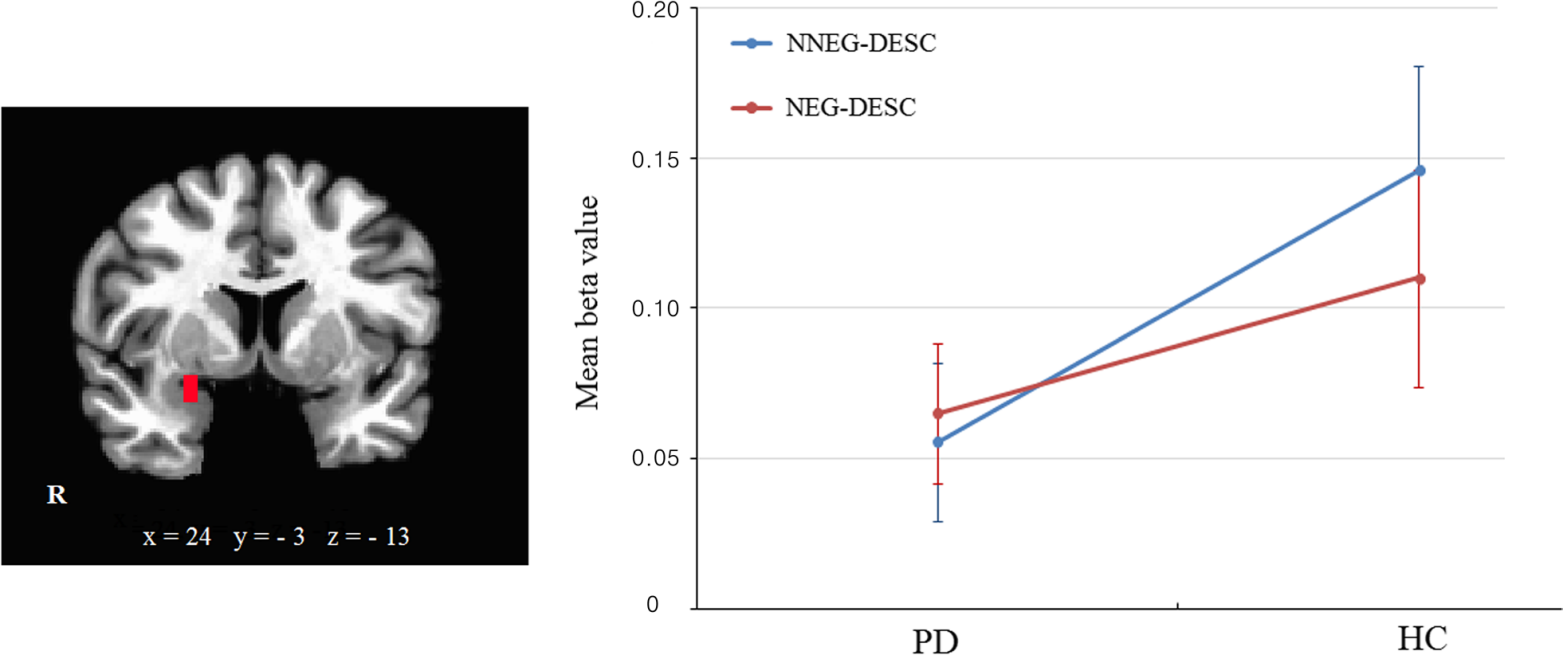
Results of ROI analysis of amygdala. Group × condition interaction shows marginal significance in right amygdala (*p* = 0.06 corrected). The simple effect analysis indicated a statistical significance was observed in HC between two conditions, but there was no statistical significance in PD. Error bars represent standard errors of the means. HC, healthy control; PD, panic disorder

ROI analyses of emotion-related responses in the NNEG-DESC and NEG-DESC conditions in the PD and HC group were conducted to explore the influences of implicit cognitive reappraisal on the amygdala. As shown in Fig. 4, there were no significant differences as a function of Group (*F*_1,49_ = 2.56, *p* > 0.05, η^2^ = 0.05) or Condition (*F*_1,49_ = 1.12, *p* > 0.05, η^2^ = 0.05 and while the group × condition interaction was marginal significance in right amygdala (*F*_1,49_ = 3.6, *p* = 0.06 corrected, η^2^ = 0.07). Simple effects analysis indicated a significant difference between the two conditions in the HC group (*t*_24_ = 2.04, *p* < 0.05), but not in the PD group (*t*_25_ = 0.15, *p* > 0.05).

### Brain-behavior relationships

We found no evidence that self-reported valence ratings were related to beta estimates drawn from the dlPFC, dmPFC, parietal cortex, and amygdale during NNEG-DESC, NEG-DESC, and NNEG-DESC vs NEG-DESC within the PD and HC groups (*p* > 0.05).

**Fig. 5:**
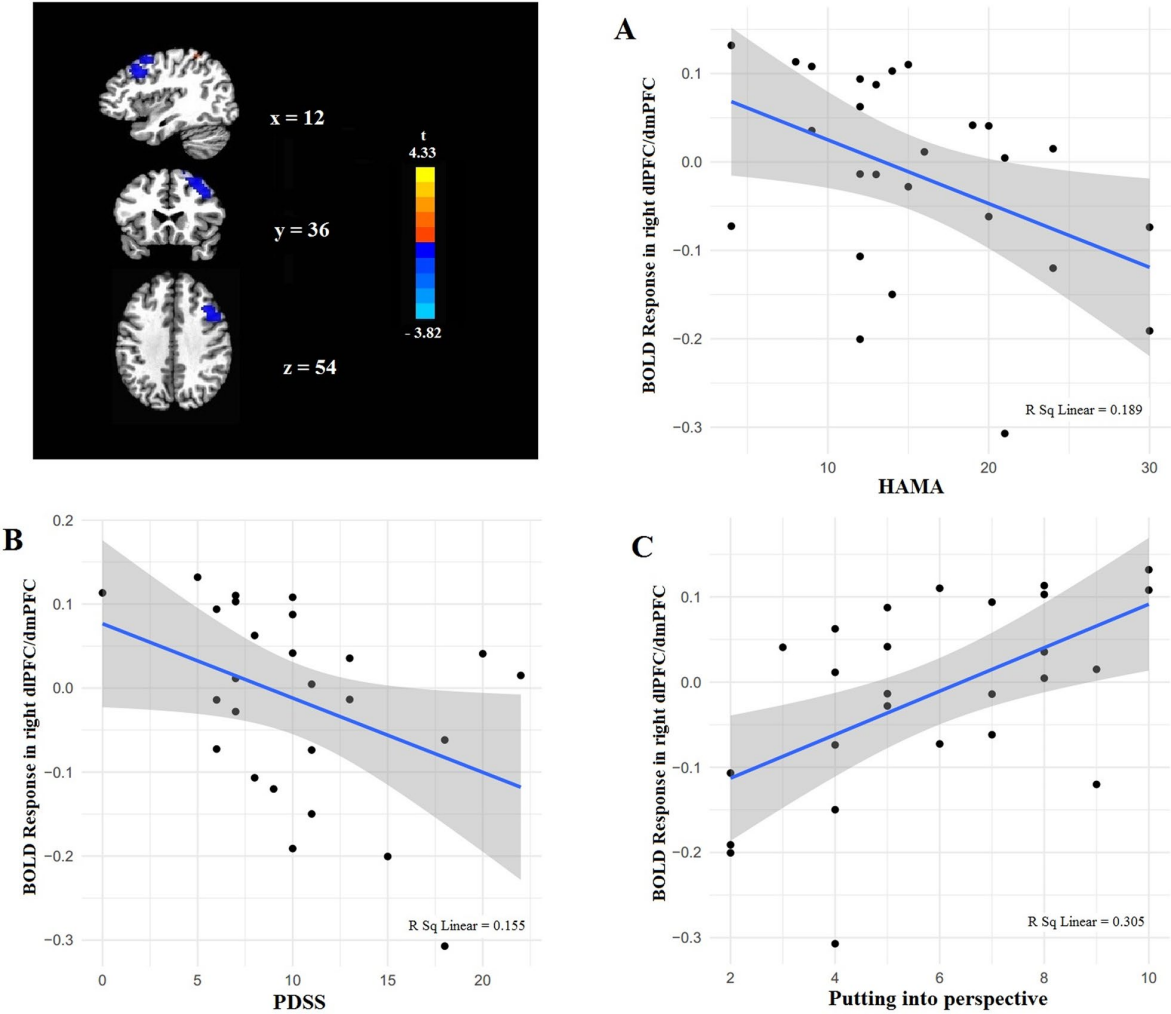
Relationships between significant clusters of activation during implicit cognitive reappraisal and symptom severity in PD. **A** Greater higher score of HAM-A showed relatively less activation in right dlPFC/dmPFC. **B** Greater higher score of PDSS showed relatively less activation in right dlPFC/dmPFC. **C** Positive relationship between the score of *putting into perspective* in CERQ and activation in right dlPFC/dmPFC. PD, panic disorder; HAM-A, Hamilton Anxiety Rating Scale; PDSS, Panic Disorder Severity Scale; CERQ, Cognitive Emotion Regulation Questionnaire; dorsolateral prefrontal cortex,dlPFC; dorsomedial prefrontal cortex, dmPFC

Relationships between symptom severity obtained from questionnaires and average brain activation during cognitive reappraisal (NNEG-DESC vs NEG-DESC) were examined for the two brain regions (right dlPFC/dmPFC and right parietal cortex) in the PD group. Owing to peak activation of dlPFC extending to dmPFC, we selected dlPFC to analyze as a whole. The results showed that those with greater higher score of HAM-A showed relatively less activation in right dlPFC/dmPFC during NNEG-DESC vs NEG-DESC (*r* = *−* 0.44, *p* = 0.026) (Fig. 5A). Similarly, PD patients with higher score of PDSS showed relatively less activation in right dlPFC/dmPFC during NNEG-DESC vs NEG-DESC (*r* = *−* 0.39, *p* = 0.046) (Fig. 5B). We additionally found a positive relationship between the score of *putting into perspective* in CERQ and activation in right dlPFC/dmPFC during NNEG-DESC vs NEG-DESC (*r* = 0.55, *p* = 0.003) (Fig. 5C). We found no relationship between brain activation and any other variables (*p* > 0.05).

## Discussion

The present study explored how PD affects brain functioning in an implicit reappraisal strategy when applied to negative picture processing. We found that the neutral/positive description decreased negative valence ratings compared to negative description when presented to healthy subjects. In contrast, there was no significant effect of prior descriptions on valence ratings for PD participants. Besides, we found interactive effects of group and condition in the right amygdala but was not affected during implicit reappraisal in the PD group, suggesting that this strategy was not an effective neural modulator of negative emotions in PD. Moreover, whole-brain results showed that PD patients exhibited less activation in the right dlPFC and right dmPFC and greater activation in parietal cortex during the NNEG-DESC vs NEG-DESC conditions, compared to HC. These findings appear to be in support of a damaged automatic top-down regulation due to prefrontal dysfunction which could not decrease responses to negative emotion in the amygdala. Finally, activation of dlPFC and dmPFC was negatively correlated with anxiety and panic severity in PD, suggesting that the failure to recruit prefrontal cortex in implicit cognitive reappraisal might be associated with the severity of anxiety and panic.

Implicit emotion regulation is considered when the subject has no subjective awareness or effort to influence the emotion regulation process but eventually attenuates the emotional response [22, 47]. In our task, subjects received a short description of the upcoming pictures and then simply watched the pictures. With this approach, subjects were given descriptions that could unconsciously influence the meaning of the forthcoming pictures, rather than leaving them to generate their own reinterpretations. In passive viewing of negative pictures, subjects simply waiting and watching, and not being instructed to moderate their emotional responses. The cognitive regulatory processes are activated and occur outside of consciousness. During this regulatory process, subjects may have an implicit goal, provoked and sustained by previous descriptions, to regulate their emotional feelings upon viewing the negative pictures. Therefore, this cognitive emotion regulation processes were effortless, uninstructed, and proceeds without awareness.

Implicit emotion regulation occurs only under the influence of certain external stimuli, which are essential for implicit emotion regulation to occur [48]. In our task, the neutral/positive interpretation preceding the negative picture induces a specific emotion in the subject, and the matching of that specific emotion with the negative picture serves as an external stimulus to elicit implicit regulatory processes. Using implicit cognitive reappraisal task with ERP, Mocaiber et al. showed that if subjects passively viewed emotional pictures produced smaller LPP amplitudes than non-emotional pictures or no significant difference was observed, it was inferred that subjects showed implicit emotion regulation [49]. Similarly, in our earlier study, this implicit cognitive reappraisal paradigm was used with fMRI to successfully explore the neural mechanisms of implicit emotion regulation in healthy individuals [23]. The process of emotion regulation described in the above study is implicit: individuals are unaware of the regulation of emotional control elicited by the stimuli on their behavior, and the regulatory process takes place largely outside of conscious awareness. These studies allow us to more reliably explore the neural basis of implicit emotion dysregulation of PD in the current study.

Reinterpretation and distancing are two main tactics studied under reappraisal [29]. It would be fruitful to discuss the differences and commonalities between reinterpretation and distancing. The distinction of these two strategies is that reinterpretation concentrates more on altering the meaning or content of the stimulus, whereas distancing emphasizes more on shifting the perspective of considering the stimulus. Both tactics are effective and favorable over other emotion regulation strategies in some contexts [29], while the two strategies were studied separately in the single study revealing that reinterpretation appears to activate more ventrolateral prefrontal cortex, whereas alienation is related to activation of parietal cortex [13]. Ochsner et al. [50] directly compared the neural basis of reinterpretation and distancing, finding increased activation in the lateral prefrontal cortex, temporal, parietal, and occipital cortex in subjects using reinterpretation and greater activation in the cingulate gyrus and parietal cortex in the group using distancing. A further between-subjects study demonstrated increased activation in brain regions including orbitofrontal cortex, frontal cortex, insula, supplementary motor areas, parietal cortex, and temporal cortex in reinterpretation compared with distancing, which more strongly enhanced activation in the parietal cortex [51]. Thus, these prior findings suggest that the prefrontal cortex involving lateral prefrontal cortex, supplementary motor areas, and orbitofrontal cortex, temporal cortex and parietal cortex were recruited in reinterpretation, while the parietal cortex was associated with distancing. Notably, distancing may be a particularly promising strategy out of the two and that the benefits of distancing motivate further investigation of the tactic [29].

According to Ochsner’s multi-level framework of implicit emotion regulation [52], implicit-controlled emotion regulation is marked with an implicit emotional regulation goal and the involvement of active control processes. In the paradigm used in our study, reinterpretation was considered as an active control that is involved in active cognitive control processes initiated by implicit goals, which is consistent with Ochsner’s implicit-control emotion regulation framework. Thus, we believe that reinterpretation may be appropriate for implicit condition.

In earlier work, we explored the neural bases of implicit emotion regulation in HC by using the implicit interpretation paradigm and demonstrated that implicit interpretation processes could recruit prefrontal areas involving prefrontal cortex involving the dlPFC and dmPFC, and parietal cortex [23]. In the current study, PD patients exhibited less activation in the dlPFC and dmPFC compared to HC, which coincides with brain regions involved in implicit cognitive reappraisal in HC and also overlaps and differs from the brain regions involved in the explicit reinterpretation. Thus, increased activation in prefrontal areas generally associated with explicit forms of reinterpretation may be also partly engaged in implicit reinterpretation.

The lack of effect of valence ratings between the NNEG-DESC and NEG-DESC conditions in the PD group suggests that when viewing the negative images proceeded by non-negative descriptions, PD subjects were not able to use implicit reappraisal to reduce subjective negative experience. Significantly, this result contrasts with Ball et al.’s report that PD individuals can successfully regulate emotions by use of explicit cognitive reappraisal strategies that require participants to deliberately transform aversive stimuli into less negative interpretation [9, 12]. As emotion regulation impairment in PD could operate at an unconscious level of cognitive appraisal [18, 28], such an explicit reappraisal paradigm may not sufficiently reveal the pathogenesis of PD.

The PD group also exhibited less activation in the right dlPFC and right dmPFC relative to HC during NNEG-DESC condition vs NEG-DESC condition. DlPFC is regarded as the core node of the regulatory network and is associated with motor suppression, working memory, reasoning, and complex cognition [12, 53]. Moreover, dlPFC has an important role in top-down cognitive control [23, 54, 55] and explicit regulation [13, 52]. Previous studies [9, 56–58] have shown hypoactivation in the dlPFC in tasks of explicit reappraisal in participant samples with anxiety and mood disorders. In a recent study, activation in dlPFC was associated with automatic cognitive top-down control using an implicit reappraisal paradigm to explore mechanisms of implicit reappraisal in healthy participants [23]. Therefore, it is possible that the impaired dlPFC in PD patients results in insufficient engagement of automatic attentional and inhibitory control, biasing patients’ attitudes toward aversive stimuli.

PD patients showed active clusters extending to dmPFC in dlPFC, but not in HC, when comparing NNEG-DESC and NEG-DESC conditions. The dmPFC is associated with elaborating the affective meaning of stimuli and monitoring emotional experiences [12] and is also related to implicit cognitive reappraisal [7]. Numerous studies from cognitive reappraisal suggest that healthy subjects could engage dmPFC to enable successful emotion regulation [6, 53]. Findings from our study, therefore, suggest that emotion regulation alterations in PD may be partially a consequence of ineffective management of monitoring and reflecting upon the implications of altered emotional stimulation in implicit cognitive reappraisal. Collectively, this results in a decreased capacity to automatically down-regulate negative responsiveness.

PD patients also showed involvement of the increased activation in right parietal cortex compared to HC in contrasting the two conditions. This cerebral region is part of the prefrontal-parietal network [7] associated with sensory information, in which response-relevant information from the top-down promotes flexible, goal-directed behavior [59]. Studies in healthy controls and individuals with pathological health anxiety have revealed that a hyper-activated parietal cortex is implicated in implicit emotional processing [60, 61]. As the prefrontal cortex and parietal cortex are the two important parts of frontoparietal network involving in implicit cognitive reappraisal [23], the increased activity of parietal cortex may be a compensatory mechanism for reduced recruitment of dlPFC and dmPFC during implicit reappraisal. This tentative interpretation suggests that dysfunction in the frontoparietal network responsible for the implicit cognitive control of negative emotions may be a characteristic feature of PD and that the hyperactivation may appear in parietal cortex as a consequence of, or to compensate for, impaired emotion regulation.

We further observed a Group × Condition interaction in the right amygdala for the HC group, but not the PD group, during implicit reappraisal. According to a prominent neurobiological model of emotion regulation, threat processing is related to signaling in limbic areas of the brain, such as the amygdala, a critical area associated with the sensing and encoding of adverse stimuli, and the successful down-regulation of this response is considered to be implicated in an increase in cognitive control of prefrontal areas [13, 62]. A recent review showed that the core concept for pathophysiology in panic disorder might be linked with disturbances in the frontal-limbic network [63]. Therefore, this result indicates that prefrontal lobes of PD patients may not sufficiently engage unconscious top-down control to decrease the activity in amygdala.

Using CERQ, we found that PD patients use less positive reappraisal and putting into perspective but more catastrophic strategies such as catastrophizing and rumination. Rumination is regarded as maladaptive and has been related to greater levels of self-reported anxiety symptoms [64], while catastrophic cognition is closely related to triggering and maintaining panic [17]. When perceiving negative images, individuals with PD give priority to self-reflection and think more about the effects of negative events, while simultaneously adapting the catastrophizing cognitive model to emphasize the negative components of emotional events to exaggerate perceptions of threat. This chain of events consequently enhances the intensity of emotional response to stimuli and triggers panic attacks and pathological anxiety [65]. Hence, this cognitive bias compels patients to implicitly choose to focus on and exaggerate threatening components when viewing aversive images, making it difficult to gather cognitive resources to adjust their emotional state to align with previous neutral/positive descriptions.

Interestingly, we observed inverse correlations between anxiety severity and dlPFC/dmPFC activation in the PD group, consistent with a prior report that anxiety severity and functional impairment are inversely associated with prefrontal activation [8]. Panic severity is negatively correlated with dlPFC/dmPFC activation during implicit reappraisal, in which the severity of panic symptoms over the last month was correlated with weaker prefrontal activity. Such findings suggest that the failure to engage the prefrontal cortex during implicit cognitive reappraisal might be associated with the severity of anxiety and panic symptoms. Furthermore, the positive relationship between putting into perspective score and activation in right prefrontal cortex was found during implicit cognitive reappraisal. The putting into perspective is the tendency to reduce the significance of a circumstance relative to other experiences [66] and is associated with the improvement of anxiety symptoms [64], implicating a promising quantitative indicator for PD treatment.

There are several limitations to this study. First, previous studies have shown regions of the prefrontal cortex associated with implicit reappraisal that could be overlapping in explicit reappraisal to modulate amygdala activation [23]. Although our results showed that dlPFC, dmPFC, and parietal cortex in PD are engaged during implicit reappraisal, these brain regions are also related to explicit reappraisal to some extent. Moreover, the PD-related changes in brain function between unconscious and conscious reappraisal were not directly compared. Further studies should elucidate the differences and commonalities of both emotion regulation strategies. Second, some patients with PD presented with co-morbid major depression, reducing our ability to classify neural engagement specific to PD. However, the high rate of co-morbidity in anxiety disorders [39] including individuals with depressive disorder or other anxiety disorders, may increase the generalizability of those with PD in community and clinical settings. Third, in this experiment we have attempted to focus our examination on differences in brain activation between PD and HC during implicit cognitive reappraisal. This study is not longitudinal in design. Longitudinal studies of the same subjects should be performed in future to further refine establish causality and neural activity changes in PD during implicit cognitive reappraisal. Fourth, as the other main reappraisal tactics for emotion regulation compared to reinterpretation, psychological distancing may be particular promising in psychopathological disorders [29], thus future studies could utilize an implicit psychological distancing strategy to more clearly differentiate the processes that are impaired in PD. Fifth, since the current study was conducted on individuals of Chinese Asians, another limitation is that these findings lack representativeness to all individuals and therefore should be generalized only to the appropriate race/demographic. Finally, there was a lack of neutral images in our experimental task that make it hard to separate the attenuation caused by non-negative description from the enhancement caused by negative description. Further studies should attempt to disentangle the two in order to clarify the neural mechanisms involved in the implicit cognitive reappraisal of PD.

## Conclusion

Patients with PD showed a different pattern of brain activation from HC when performing an implicit cognitive reappraisal task. Specifically, patients were not able to recruit some of the prefrontal regions (i.e., dlPFC and dmPFC) to modulate emotional responses in the amygdala, suggesting that emotional dysregulation in PD is likely the result of compromised top-down, automatic regulation of negative emotions. These results provide a valuable target for future research evaluating therapeutic interventions for PD that rely on implicit reappraisal (e.g., cognitive behavioral therapy) [67, 68] or neuromodulatory interventions (e.g., transcranial magnetic stimulation) [69]. A negative relationship between severity of anxiety and panic and activation of right dlPFC and dmPFC in the present study demonstrate that the failure to engage prefrontal cortex during implicit cognitive reappraisal might be associated with the severity of anxiety and panic symptoms. This study sheds new light on the neural dysfunction underlying PD during emotion regulation, highlighting the important role of functional changes in dlPFC and dmPFC that could be useful in understanding the neuropathological mechanisms underscoring PD and how to treat it.

## Data Availability

The datasets used and/or analyzed during the current study are available from the corresponding author on reasonable request.

## Abbreviations

PD: Panic disorder
HC: Healthy controls
dlPFC: Dorsolateral prefrontal cortex
dmPFC: Dorsomedial prefrontal cortex
ERPs: Event-related potentials
LPP: Late positive potential
DSM-5: Diagnostic and statistical manual of mental disorders, 5th Edition
HAM-A: Hamilton Anxiety Rating Scale
HAM-D: Hamilton Depression Rating Scale
PASS: Panic-Associated Symptom Scale
PDSS: Panic Disorder Severity Scale
CERQ: Cognitive Emotion Regulation Questionnaire
NEG-DESC: Negative descriptions preceding negative pictures condition
NNEG-DESC: Non-negative descriptions preceding negative pictures condition
ANOVA: Analysis of variance
BOLD: Blood oxygenation level-dependent
FOV: Field of view
AFNI: Analysis of Functional NeuroImages
ROI: Regions-of-interest
